# To the Editors of the Medical and Physical Journal

**Published:** 1810-06

**Authors:** J. Howe


### *To the Editors of the Medical and Physical Journal.*

GENTLEMEN,

IN your Journal for April, there is a question put to me, Whether a sufficient and steady pressure cannot be made upon the artery above the clavicle, so as effectually to stop the current of blood in the axillary artery? It has frequently been done, and is what I believe to have been universally adopted, in the numerous instances of amputation at the shoulder-joint, that have come to my knowledge. With respect to the assertion following the question, I doubt if it will be found to be unexceptionable. Let us suppose a surgeon of a sloop of war meets with a case requiring amputation at the shoulder-joint; to the purser, who generally assists him, he would trust for compressing the artery; after having adjusted the key (or any other instrument he may choose to adopt) upon the artery, what a situation the surgeon would be in, as soon as he has divided the artery, should the purser happen to faint, or not to compress the artery, and no surgical assistance near him. This, although embarrassing, it may be asserted, would not much impede the operation, as the surgeon would compress the artery with one hand, whilst he takes up the bleeding vessel with a tenaculum; yet, although this *may* be done, I myself should prefer guarding against it, by tying the artery. In my letter in your Journal of February, I did not recommend tying the artery in the axilla, as preferable to compressing the artery above the clavicle; I only mentioned it in describing the manner in which I had operated. I will now point out my reasons for having done so. I have previously mentioned that there were only two medical officers present at the operations, and I preferred the surgical assistance being directed to the supporting and moving the shattered limb, in assisting to tying the arteries, &c. in preference to the compressing the artery. I also wished the patient to be kept as quiet as possible during the operation, without much holding; and as the pressure above the clavicle when made sufficiently firm, completely to stop the current of blood, is extremely painful, I felt pleased in having it in my power to prevent this, as the motion which the patient gives to the shoulder, in trying to get rid of the pressure at the clavicle, unless he has the greatest fortitude, must be extremely troublesome, where the operator is not surrounded with numerous hands to keep the patient firm.

I am, &c.

J. HOWE.

*May* 8, 1810.

